# Assistive Mobile Application for Fire Emergency Evacuation of Visually Impaired People

**DOI:** 10.3390/s26051572

**Published:** 2026-03-02

**Authors:** Adrian Mocanu, Camelia Avram, Dan Radu, Ioan Valentin Sita, Adina Astilean

**Affiliations:** Automation Department, Technical University of Cluj-Napoca, 400114 Cluj-Napoca, Romania

**Keywords:** fire emergency evacuation, dynamic routing, mobile application, software applicatio, impaired people

## Abstract

The emergency evacuation of visually impaired individuals during fire incidents presents critical challenges that require innovative technological solutions. While existing evacuation systems provide static route guidance, they fail to adapt dynamically to evolving fire conditions, blocked passages, or dangerous zones in buildings with multiple routes and exits. This paper presents a comprehensive implementation of a mobile application built with Flutter/Dart that addresses these limitations by enabling real-time, dynamic route computation based on live sensor data. The presented system operates in a decentralized manner, performing all critical computations on-device to ensure its functionality even when some parts of the building infrastructure fail. A dynamic route calculation modified Dijkstra’s algorithm was implemented on each user’s phone for guidance. If initial path adjustments are needed, they are computed from sensor data to evaluate fire evolution and other relevant factors, including the user’s current position and crowd congestion. An audio–visual interface was designed to provide navigation instructions and to help users follow safety routes efficiently. Field testing with visually impaired participants demonstrated significant improvements in evacuation efficiency, with shorter evacuation times than traditional static guidance approaches. The system architecture complies with international fire safety standards while maintaining user privacy through a no-tracking design philosophy. This work contributes to both theoretical advances in adaptive evacuation algorithms and practical insights for deploying assistive technologies in emergency scenarios.

## 1. Introduction

### 1.1. Motivation and Background

Fire emergencies in buildings represent one of the most severe threats to human life, with statistics from the European Fire Safety Alliance indicating over 5000 fatalities and 50,000 injuries annually across EU member states alone [1]. For visually impaired individuals, these risks are also dramatically amplified. Studies by the National Institute of Standards and Technology demonstrate that visually impaired persons trapped on smoke-filled floors experience significantly higher rates of disorientation, extended evacuation times, and elevated mortality risks compared to sighted individuals [2].

Traditional building evacuation systems rely heavily on visual cues—illuminated exit signs, floor plans, and directional arrows—that become ineffective or inaccessible when smoke obscures visibility or individuals cannot perceive them. While tactile indicators and audible alarms provide some assistance, they offer only generic warnings without personalized, location-aware guidance. Furthermore, these static systems cannot adapt to dynamic fire conditions in which initially safe routes are blocked by flames, smoke, or structural failures.

The proliferation of smartphones presents an unprecedented opportunity to deliver personalized, real-time evacuation guidance directly to individuals in emergencies. Modern mobile devices possess multiple sensors (accelerometers, gyroscopes, magnetometers), wireless communication capabilities (Wi-Fi, Bluetooth), and substantial computational power—sufficient to perform complex pathfinding calculations locally without dependence on external servers or building infrastructure that may fail during emergencies.

### 1.2. Research Gap and Challenges

Despite extensive research in indoor navigation and emergency evacuation systems, several critical gaps remain:

1. **Dynamic Adaptation**: Most existing systems compute evacuation routes based on pre-planned paths or static building layouts. They lack mechanisms to incorporate real-time sensor data reflecting fire spread, smoke accumulation, and corridor blockages.
2. **Decentralized Processing**: Many proposed solutions rely on centralized servers or cloud computing for route calculation, creating single points of failure when network infrastructure becomes compromised during emergencies.
3. **Multi-Factor Optimization**: Traditional shortest-path algorithms consider only geometric distance, neglecting crucial factors such as smoke density, crowd congestion, user mobility characteristics, and route robustness to potential blockages.
4. **Indoor Positioning Accuracy**: GPS signals are unreliable indoors, which requires alternative positioning technologies. Wi-Fi fingerprinting and BLE beacons offer solutions, but require careful implementation to achieve sufficient accuracy for turn-by-turn navigation.
5. **Implementation Practicality**: The academic literature often presents theoretical frameworks without addressing practical implementation challenges such as mobile platform compatibility, battery efficiency, real-time performance constraints, and user interface accessibility.
6. **User Speed Consideration**: Evacuation routing should account for individual walking speeds, especially for visually impaired users who may navigate more slowly or require additional time for tactile way-finding.

### 1.3. Contributions

This paper addresses these gaps through the following contributions:

1. **Mobile-First Architecture**: We present a complete Flutter/Dart implementation designed for cross-platform deployment (iOS, Android) with all critical computations performed on-device for resilience against infrastructure failures.
2. **Inclusive Design Approach**: Mobile applications are reliable for users with a range of disabilities, including visual impairments, hearing impairments, and mild cognitive impairments.
3. **Adaptive Dijkstra’s Algorithm**: We propose a modified Dijkstra’s algorithm that dynamically adjusts edge weights based on real-time sensor data, incorporating fire proximity penalties, smoke density factors, crowd congestion metrics, and user-specific walking speeds.
4. **Wi-Fi RSSI Positioning System**: We integrate Wi-Fi signal strength measurements for indoor localization using trilateration and filtering techniques to achieve a positioning accuracy suitable for corridor-level navigation.
5. **Real-Time Route Recalculation**: The system continuously monitors sensor broadcasts and automatically triggers route recalculation when blockages are detected on the current path, with seamless audio guidance updates. Recalculation is optimized to maintain real-time instructions.
6. **Robustness Optimization**: Beyond finding the shortest safe path, our algorithm evaluates route robustness—ensuring selected paths have viable alternatives if any single corridor becomes blocked.
7. **Empirical Validation**: We present experimental results from field testing with visually impaired participants, demonstrating measurable improvements in evacuation efficiency and user confidence.

### 1.4. Paper Organization

The remainder of this paper is organized as follows: Section 2 reviews state-of-the-art approaches in indoor positioning, emergency evacuation systems, and assistive technologies. Section 3 describes the system architecture and mobile application design. Section 6 details the Wi-Fi RSSI-based positioning methodology. Section 4 presents the modified Dijkstra algorithm including pseudocode and a complexity analysis. Section 7 discusses implementation considerations in Flutter/Dart. Section 8 presents an implementation of the communication network using ns-3 and Quality of Service analysis of it. Section 9 presents the experimental results and performance evaluation. Section 10 concludes with future research directions.

## 2. State of the Art

In the work related to the evacuation of visually impaired people from buildings that are on fire, we consider an entire pipeline, from sensing and localization, through environment representation as a topological graph derived from a floor plan or map, to real-time, multi-objective route optimization, followed by a multimodal interface (audio/haptic/visual) and validation via simulation. For users with visual impairments, the literature reports a consistent shift from single-tech solutions to hybrid systems (BLE/Wi-Fi/UWB combined with IMU, vision, ultrasonic, and mobile applications), with a strong focus on operational robustness, the consistency of instructions, and scalability in public spaces, with recent review papers serving as helpful reference points for classifications, standards, and current research gaps [3,4,5,6].

Over the past decade, digital evacuation support has shifted from fixed infrastructure to devices in everyone’s hands [7]. The smartphone has emerged as a guidance node: it can fuse indoor positioning, scene perception (computer vision and lightweight AR), a building’s digital representation, and adaptive messaging to deliver personalized routes and instructions [8]. Multiple studies report that mobile applications can provide dynamic personalized guidance and various UI modalities [7,9,10,11]. This technical convergence aligns with a change in people’s attitudes. Interviews report a strong willingness to rely on phones for instructions in buildings that are on fire, suggesting that the mobile channel provided by the application offers credible routes, remains resilient to localization errors, and communicates clearly [12,13,14]. Beyond algorithmic pathfinding, there are two valuable practical premises: evacuation time is jointly determined by individual heterogeneity and exit congestion, and minute-level improvements in response times translate into meaningful life safety gains [15,16]. The study by [17] develops an evacuation time estimation model for large buildings that explicitly parameterizes pedestrian characteristics and real-time exit crowding, thereby motivating congestion-aware rerouting at the handset level. In parallel, Jaldell [18] quantifies the value of time in fire and rescue contexts, showing that a faster response leads to measurable life safety benefits. Crucially for accessibility, the article by [19] presents the distinct movement patterns of blind and visually impaired evacuees under fire conditions, implicating lower effective speeds, different pre-movement behaviors, and a stronger dependence on non-visual cues.

For deaf and hard-of-hearing people, empirical work in sleep contexts shows that low-frequency alarm signals (around 520 Hz) and tactile notifiers (bed/pillow shakers) are effective in awakening and alarming [20]. Although the bedroom use case is specific, the broader principle of combining low-frequency audio with robust tactile/visual notifications generalizes to mobile evacuation apps (e.g., strong vibration patterns plus text and pictograms), particularly for users with residual hearing. Beyond alerting, newer mobile sound recognition applications with interactive ML can translate critical auditory events (sirens, fire alarms, spoken announcements) into tactile/visual cues during pre-movement, thereby reducing uncertainty before route guidance begins [21,22].

Mobility limitations introduce different constraints. Reviews focused on functional limitations in high-rise buildings survey vertical evacuation aids, defend-in-place strategies, refuge areas, and the circumstances under which evacuation elevators can be used safely [23,24,25]. A mobile app can serve as a personal plan broadcaster that aligns users, assistants, and facility teams and as an interface to refuge systems while filtering building graphs to identify barrier-free routes. Regarding inclusive evacuation, beyond visual impairment, review articles map existing solutions and gaps for groups with functional limitations, including high-rise buildings, and cover aspects ranging from human behavior to infrastructure provisions, such as refuge areas and evacuation elevators. A recurring conclusion is that accessibility criteria (e.g., avoiding stairs, slopes, and width constraints; and providing multimodal signage) are poorly standardized in routing models, despite being essential for realistic evacuation times [24].

For indoor navigation targeting users with visual impairments [3], foundational work has combined UWB/BLE infrastructure with topological representations and dynamic programming algorithms, thereby validating smartphone-based guidance. The GuideBeacon BLE line [26] remains a representative example in terms of the cost–coverage trade-off in large buildings. In parallel, infrastructure approaches based on mobile computer vision with IMU fusion are advancing, while systems such as VISA [27] integrate global navigation and proximity perception into a single application. Recent evaluations suggest that, in the presence of crowding or smoke, the temporal consistency of the position estimate and the quality of instructions matter more than absolute metric accuracy [28]. Building on infrastructure-assisted navigation, Ref. [29] presents SUGAR, a UWB-based smartphone system that fuses high-precision indoor positioning with a path-planning algorithm, demonstrating feasibility through a functional prototype tested with a blind user and highlighting UWB’s accuracy and its relatively light deployment in complex interiors. In contrast, Ref. [30] advances an RFID-based architecture that maps building blueprints, localizes the user, and performs obstacle-aware guidance for visually impaired and elderly users.

In recent years, the literature has converged on the idea that the smartphone can act as the primary guidance interface for daily activities [27,31] or during evacuations, as it enables personalized, multimodal, real-time delivery of instructions, even when static signage is compromised by smoke or darkness [32,33]. A particularly experimental result comes from the study dedicated to SVGES (Smartphone Voice-Guided Evacuation System) [7]: in a university basement, all 26 participants (100%) successfully evacuated with the application enabled, compared to 58% without it, a difference attributed primarily to the voice instructions, which override visual ambiguity under smoke conditions. The system relies on Bluetooth beacons associated with detectors, pre-designed routes compliant with relevant codes, and audio/graphical notifications on the phone, thereby avoiding a strict reliance on high-accuracy positioning when such estimates become unstable. Despite this, tactile feedback remains valuable and provides users with confidence [34,35].

On the occupant-acceptance side, qualitative interview studies indicate that people who carry their smartphones with them during evacuation expect personalized information (e.g., “the safest route in this moment is…”) and prefer short, clear messages delivered with minimal delay. These findings support an interface design based on step-by-step, minimalist, spatially synchronized audio instructions and confirm the usefulness of redundant channels (text and pictograms) when hearing is not impaired [12].

Recent research increasingly shifts from occupant-only guidance to collaborative fire emergency response, where evacuation routing is coupled with information exchange and responder workflows. For example, the CORE system explicitly supports indoor navigation and information sharing among occupants, firefighters, and facility managers, enabling person-to-person rescue, evacuation, and access to resources for suppression [36]. Complementary work also integrates real-time evacuation with rescue-oriented actions, highlighting the need to align automated guidance with operational constraints and responder priorities during dynamic incidents [37]. In addition, BIM-centred emergency platforms increasingly fuse hazard simulation and building semantics to provide updated situational information, which is crucial for coordinating evacuation decisions with on-scene responses [38].

Emergency wayfinding is strongly shaped by acute stress and uncertainty, which can alter perception, attention, and decision-making; recent syntheses formalize these effects and report strong compliance effects for salient cues and social influence in emergencies [39]. Beyond general population behaviour, controlled experiments show that stress can measurably degrade navigation efficiency and strategy choice, underscoring the importance of designing guidance that minimizes cognitive burden [40]. For impaired users, recent assistive navigation studies highlight that guidance modality and message complexity can introduce additional cognitive load; accordingly, systems should prioritize concise, unambiguous prompts and low-cognitive load interaction patterns, especially when conditions are time-critical [41]. Decision-making research in fire-like VR scenarios further indicates that smoke can alter exit choice behavior and the relative weight of familiarity/social cues, reinforcing that smoke-aware guidance and modeling are critical for fire evacuation validity [42].

Recent post-disaster communication research demonstrates rapidly deployable ad hoc networking approaches that enable device-to-device exchange without relying on intact infrastructure—an important direction for decentralized, field-ready systems [43]. In parallel, responder-focused mobile tools combine tracking (e.g., IMU/RFID or similar) with geo-referenced Points of Interest, supporting coordination and situational awareness during indoor missions where GPS fails [44].

The current landscape effectively legitimizes the smartphone as the final guidance node in evacuation: from solid experimental results (e.g., SVGES) and frameworks that reroute on the order of seconds to platforms such as EVAGUIDE/eVACUATE. Based on this, we explicitly commit in this paper to the following pipeline: map–regulation-compliant graph–accessibility-aware cost function (smoke, congestion, stairs/slopes, decision-point density)–algorithm routing with rerouting–audio and video instructions directly on the smartphone. Moreover, for users with visual impairments, the voice channel remains central. In contrast, visual feedback is a natural extension, enabling a mobile application to close the sensor optimization feedback loop that enables visual feedback.

## 3. The System Architecture

**Figure 1:**
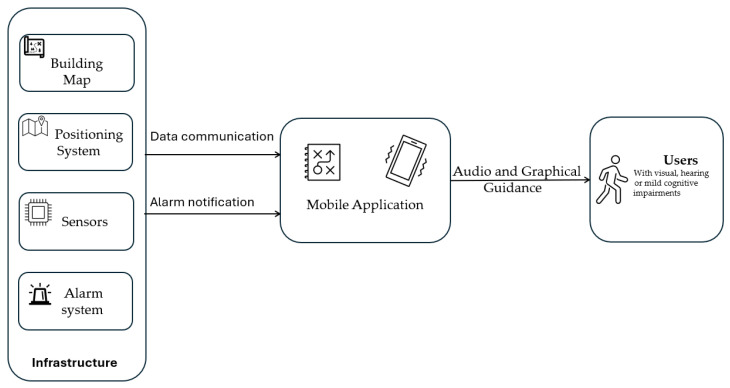
Block diagram of the evacuation system.

The proposed system employs a decentralized architecture in which critical evacuation computations are performed entirely on the user’s mobile device. This design ensures operational continuity even when building network infrastructure fails. The system integrates real-time environmental monitoring with personal navigation assistance to guide visually impaired users safely during fire emergencies. The block diagram of the presented system is shown in Figure 1.

### 3.1. Main Components of the System

The guided evacuation system consists of five interconnected components. The **Fire Alarm Sensor Network** is a distributed sensor network that monitors building conditions (smoke, temperature, fire) in real time and broadcasts hazard data. The **Indoor Positioning System** tracks the user’s location within the building using Wi-Fi RSSI and BLE beacons, as detailed in Section 6. The **Route Finding Engine** serves as the computational core that computes optimal evacuation paths from the building graph and dynamic hazard data, as further described in Section 4. The **mobile application** provides the user interface with audio and haptic guidance, ensuring accessibility for visually impaired individuals. Finally, the **Decision Support System** serves as an intelligent layer that evaluates potential routes based on safety, efficiency, and reliability.

### 3.2. Decision Support System

The Decision Support System (DSS) acts as the coordinator of the evacuation assistant. It continuously evaluates available paths via **Route Evaluation**, selecting the optimal route by balancing distance, estimated travel time, and safety factors. The DSS also incorporates **Risk Assessment** reasoning techniques to handle uncertainty, assigning risk penalties to areas with reported smoke or crowding. Designed for **Real-Time Operation** with low latency, the system processes sensor updates and re-evaluates routes in under 200 ms, ensuring timely guidance.

### 3.3. Redundant Physical Cues

To enhance safety and comply with accessibility standards (ISO 21542 [45]), the digital system is complemented by physical guidance aids. For this, 30 mm PVC tactile strips with a ribbed profile (R9 anti-slip rating) are installed 250 mm off the wall along primary evacuation routes. The mobile application integrates with these physical cues by instructing users to “follow the tactile strip” when their lateral deviation exceeds 0.6 m, as detected by the smartphone’s accelerometer.

### 3.4. Route Robustness and Reliability

The system ensures a robust performance through several mechanisms. Multiple alternative routes are **pre-calculated** from key decision points to provide rapid switching if the primary path is blocked. When obstacles or hazards are detected, the system immediately triggers a **Dynamic Recalculation** of the route. Furthermore, **Data Validation** ensures that sensor data is validated through redundancy to prevent false alarms from triggering incorrect routing decisions.

### 3.5. Security and Privacy Architecture

The system is designed with a “privacy-by-design” approach. It operates without continuous tracking or GPS, and does not transmit personal data or user location to any central server. The mobile application listens to broadcast hazard alarms but remains silent regarding the user’s position, ensuring that sensitive location data never leaves the device. Communication integrity is maintained using signed BLE frames (16-bit CRC and 128-bit CBC-MAC) with rotating keys.

### 3.6. Fire Detection and Alarm Prototype

**Figure 2:**
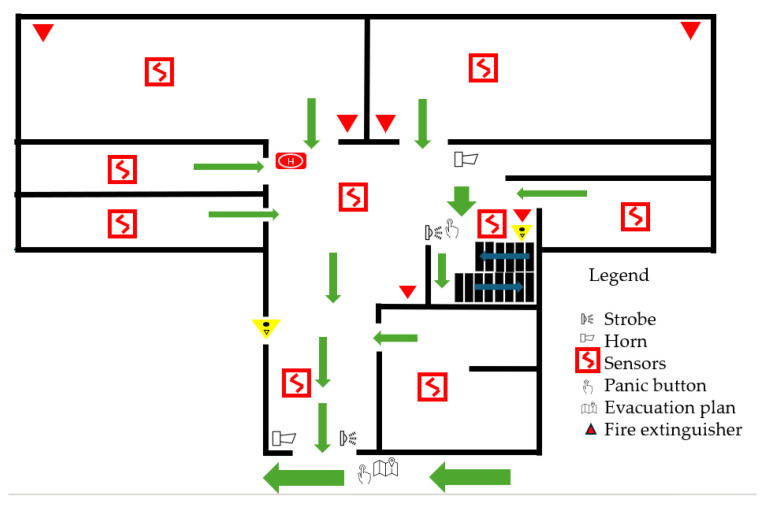
Fire detection prototype for one sector of the building.

The fire detection and alarm prototype was implemented to develop and evaluate the proposed application. This integrates sensors and actuators representative of a conventional fire detection system and is presented in Figure 2. The sensing subsystem for each sensor consists of an MQ-2 smoke sensor, a DS18B20 digital temperature sensor, and an infrared flame detector. All sensors are connected to a NodeMCU microcontroller, which serves as the alarm zone processing unit. Audible–visual notification devices (horn–strobe units) were installed on each level and connected to a NodeMCU through a dual-relay module. All NodeMCU units for individual alarm zones are interconnected using LogiLink USB extenders, enabling long-distance communication and centralized power distribution via USB hubs. Within the building, each room is defined as an individual alarm zone. At the same time, corridors are subdivided into multiple zones based on distance to account for the detectors’ coverage limitations. Fire-resistant FTP cables compliant with IEC 60331 were installed to ensure continued data transmission for a specified duration (60, 90, or 120 min) under direct fire exposure.

## 4. Dynamic Evacuation Routing Algorithm

### 4.1. Problem Definition

The emergency evacuation routing problem aims to determine the optimal path from the user’s current position to the nearest safe exit, subject to dynamic constraints arising from fire spread, smoke accumulation, and corridor blockages. The traditional Dijkstra’s algorithm finds the shortest path in a static graph. Our modification makes it work for dynamic evacuation scenarios:

1. **Dynamic Edge Weights**: Graph edge costs change continuously as sensors detect evolving fire conditions
2. **Multi-Objective**: Routes must balance distance, safety, congestion, and robustness simultaneously.
3. **User-Specific**: Routing should account for individual walking speeds and mobility characteristics.
4. **Time-Critical**: Routes must be computed within sub-second time frames for real-time guidance.
5. **Robustness**: Selected routes should maintain viability even if individual corridors become blocked.

**Figure 3:**
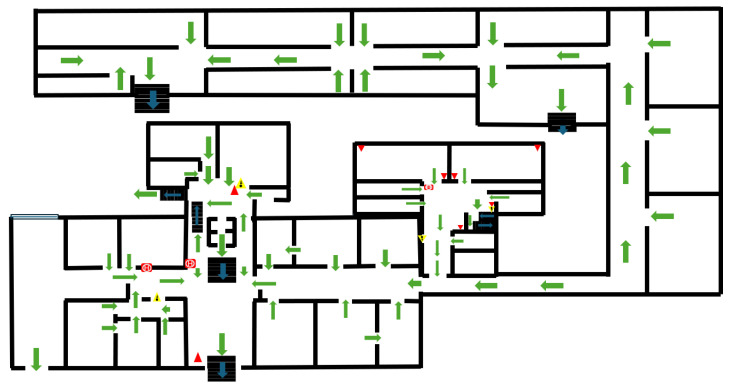
Representation of the evacuation plan.

In Figure 3, an example of an evacuation plan is represented for an existing three-story office building. Green arrows indicate exit signs and the main path.

**Figure 4:**
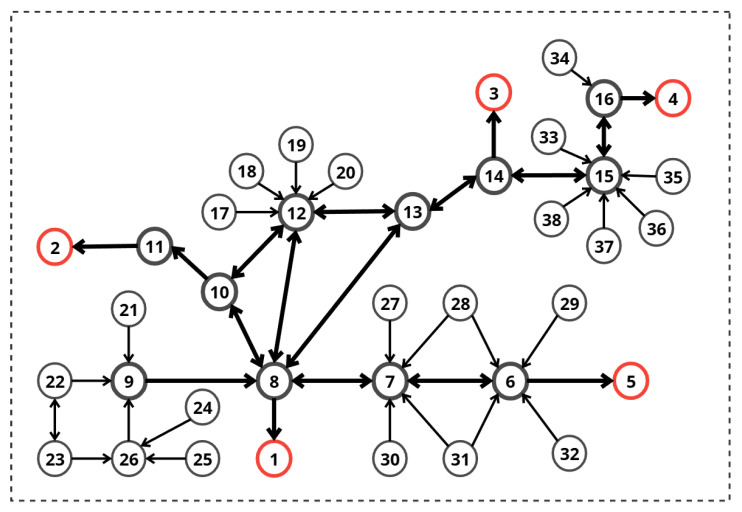
Building graph.

A corresponding weighted directed graph of the evacuation plan presented above (Figure 4) was constructed as follows.

The structure of the building is represented as a weighted directed graph G=(V,E) where **the vertices** (*V*) represent key locations, including corridor intersections, room entrances/exits, staircase landings, building exits (goal nodes), and the user’s current position (source node).

- $V=\{v_{1},v_{2},\ldots ,v_{n}\}$ is the set of *n* nodes that represent locations in the building.
- E⊆V×V is the set of edges that represent traversable corridors or paths.
- $V_{exit}\subset V$ is the subset of exit nodes.
- $V_{blocked}\subset V$ is the dynamically updated set of blocked nodes.

Each node $v_{i}\in V$ has the following properties:

(1) $$v_{i}=\left\{id_{i},{\mathrm{name}}_{i},{\mathrm{isExit}}_{i},{\mathrm{isBlocked}}_{i}\right\}$$

where ${\mathrm{isExit}}_{i},{\mathrm{isBlocked}}_{i}\in \left\{\mathrm{true},\mathrm{false}\right\}$ are Boolean flags.

Each edge $e_{ij}\in E$ connecting the nodes $v_{i}$ and $v_{j}$ has

- $d_{ij}$: base distance (constant physical distance in meters).
- $c_{ij}\left(t\right)$: current cost at time *t* (dynamically updated).

### 4.2. Dynamic Cost Model

The dynamic cost model adjusts the edge weights in response to real-time environmental conditions. The cost function incorporates multiple penalty factors.

For each node $v_{i}$, sensor data $S_{i}\left(t\right)$ at time *t* include

(2) $$S_{i}\left(t\right)=\left\{\sigma_{i}\left(t\right),\kappa_{i}\left(t\right),\phi_{i}\left(t\right)\right\}$$

where

- $\sigma_{i}\left(t\right)\in \left[0,1\right]$: normalized smoke level.
- $\kappa_{i}\left(t\right)\in \left[0,1\right]$: normalized crowding level.
- $\phi_{i}\left(t\right)\in \left\{\mathrm{true},\mathrm{false}\right\}$: fire presence indicator.

#### 4.2.1. Dynamic Edge Cost Function

The cost of the edge $e_{ij}$ at time *t* is computed as

(3) $$c_{ij}\left(t\right)=d_{ij}\cdot P_{\mathrm{smoke}}\left(e_{ij},t\right)+P_{\mathrm{crowd}}\left(e_{ij},t\right)+P_{\mathrm{fire}}\left(e_{ij},t\right)$$

Here, each penalty function is defined below.

#### 4.2.2. Smoke Penalty

The smoke penalty multiplies the base distance by a factor proportional to the average smoke level along the edge.

(4) $$P_{\mathrm{smoke}}\left(e_{ij},t\right)=1+\alpha \cdot {\bar{\sigma }}_{ij}\left(t\right)$$

where

(5) $${\bar{\sigma }}_{ij}\left(t\right)=\frac{\sigma_{i}\left(t\right)+\sigma_{j}\left(t\right)}{2}$$

And α=5.0 is the smoke amplification factor. This means that heavy smoke (σ=1.0) can increase the effective distance by up to 6 times.

#### 4.2.3. Crowding Penalty

The crowding penalty adds an absolute time cost based on congestion:

(6) $$P_{\mathrm{crowd}}\left(e_{ij},t\right)=\beta \cdot {\bar{\kappa }}_{ij}\left(t\right)$$

where

(7) $${\bar{\kappa }}_{ij}\left(t\right)=\frac{\kappa_{i}\left(t\right)+\kappa_{j}\left(t\right)}{2}$$

And β=10.0 is the delay factor in crowding (in seconds). Maximum crowding (κ=1.0) adds 10 s to the traversal time.

#### 4.2.4. Fire Penalty

When fire is detected at either endpoint of an edge, an extreme penalty is applied:

(8) $$P_{\mathrm{fire}}\left(e_{ij},t\right)=\left\{\begin{array}{cc} inf & \mathrm{if}\;\phi_{i}\left(t\right)=\mathrm{true}\;\mathrm{or}\;\phi_{j}\left(t\right)=\mathrm{true}\\ 0 & \mathrm{otherwise} \end{array}\right.$$

This penalty makes fire-affected edges extremely costly while keeping them theoretically traversable, allowing the algorithm to find alternative routes without requiring special handling of infinite weights.

## 5. Dijkstra’s Algorithm for Evacuation Routing

### 5.1. Standard Dijkstra’s Formulation

The shortest path from the source node *s* to the target node *t* minimizes the total cost of the path as follows:

(9) $$\min_{P\in P(s,t)}\sum_{e_{ij}\in P}c_{ij}\left(t\right)$$

where P(s,t) is the set of all paths from *s* to *t*.

The algorithm maintains the following: the minimum distance from the source to node *v* (d[v]), the predecessor of node *v* on the shortest path (π[v]), and a priority queue of unvisited nodes ordered by distance (*Q*). To find the best exit among multiple alternatives, the algorithm described in Algorithm A2 was used.

### 5.2. Proposed Dynamic Cost Management Algorithm

The system continuously updates edge costs based on the incoming sensor data. The proposed algorithm, described in Algorithm A3, combines continuous environmental monitoring, dynamic path recomputation, and user-centric direction guidance. The complete evacuation system operates in a continuous monitoring loop as depicted in Algorithm A4.

## 6. Wi-Fi RSSI-Based Indoor Positioning

Accurate user location is essential for computing evacuation routes from the current position to safe exits. The system implements Wi-Fi-based indoor positioning using Received Signal Strength Indicator (RSSI) measurements from multiple access points.

The positioning algorithm estimates the user coordinates (x,y) by measuring the signal strength of n≥3 access points with known positions $\left(x_{i},y_{i}\right)$. The relationship between RSSI and distance follows the log-distance path loss model:

(10) $$RSSI_{i}=RSSI_{0}-10\cdot \eta \cdot \log_{10}\left(\frac{d_{i}}{d_{0}}\right)+X_{\sigma }$$

where

- $RSSI_{i}$: Measured signal strength from access point *i* (dBm).
- $RSSI_{0}$: Reference signal strength at distance $d_{0}=1$ m.
- η: Path loss exponent (typically 2–4 for indoor environments).
- $d_{i}$: Distance to the access point *i*.
- $X_{\sigma }$: Zero-mean Gaussian noise representing multipath interference.

The Flutter application continuously scans for Wi-Fi signals and applies weighted least squares optimization to estimate position as described in Algorithm A1.

**Weighted Least Squares**: Minimizes the weighted residual error to find the optimal user position:

(11) $$\left(x,y\right)=\arg min_{x,y}\sum_{i=1}^{n}w_{i}{\left(\sqrt{{\left(x-x_{i}\right)}^{2}+{\left(y-y_{i}\right)}^{2}}-d_{i}\right)}^{2}$$

where $\arg min_{x,y}$ finds the (x,y) coordinates that minimize the sum, $\left(x_{i},y_{i}\right)$ are known AP positions, $d_{i}$ are estimated distances from RSSI, and $w_{i}$ are confidence weights.

The estimated position (x,y) is mapped to the nearest graph vertex $v_{source}\in V$ using

(12) $$v_{source}=\underset{v\in V}{arg min}{∥(x,y)-(v.x,v.y)∥}_{2}$$

where ${arg min}_{v\in V}$ finds the vertex *v* that minimizes the expression, v.x and v.y are the coordinates of the vertex *v* in the building graph, and ${∥\cdot ∥}_{2}$ denotes the Euclidean distance (L2 norm): $\sqrt{{\left(x-v.x\right)}^{2}+{\left(y-v.y\right)}^{2}}$.

Position updates trigger route recalculation when

- User moves >5 m from previous position.
- Estimated position deviates >3 m from planned route.
- Position accuracy drops below threshold (σ>5 m).

**Accuracy**: With 4–6 access points, the system achieves 2–4 m positioning accuracy in typical building environments, sufficient for corridor-level routing. Kalman filtering could be implemented in the future to improve jitter reduction and reduce false recalculations caused by RSSI variability.

## 7. Flutter/Dart Implementation

The evacuation routing system is implemented in Dart and the Flutter framework. This technology stack choice provides several critical advantages for a real-time, safety-critical application. Flutter provides cross-platform development (iOS/Android) with native performance through Dart-to-ARM compilation [46]. Its reactive UI and built-in accessibility support (TalkBack/VoiceOver integration) make it ideal for deploying assistive technology. Some of the advantages of Dart and Flutter are as follows:

- **Performance and AOT Compilation:** Dart compiles to native machine code (ARM/x86) for mobile devices (Ahead-Of-Time compilation). This ensures a consistent, low-latency performance, crucial for recalculating evacuation routes in real time as environmental conditions change.
- **Strong Typing and Null Safety:** The Dart type system, with its sound null safety, significantly reduces the risk of runtime errors (such as null pointer exceptions) that could cause the application to crash during an emergency.
- **Asynchronous Processing:** Dart’s Future and Stream APIs allow for the efficient handling of asynchronous sensor data updates without blocking the main execution thread, ensuring that the user interface remains responsive.
- **Cross-Platform Deployment:** The Flutter framework enables the deployment of a single codebase to both iOS and Android platforms, ensuring broad accessibility for all building occupants.

The application uses three isolates (Dart’s lightweight threads): **Main Isolate**: UI rendering; **Positioning Isolate**: continuous sensor processing; **Routing Isolate**: pathfinding and sensor monitoring without blocking the UI. Isolates communicate via message passing, ensuring that pathfinding latency does not affect UI responsiveness.

### 7.1. User Interface

Within the **Main Isolate** (UI), state management is handled natively through the StatefulWidget lifecycle, ensuring efficient updates to UI components. For auditory feedback, the system integrates the flutter_tts package, providing synthesized text-to-speech capabilities to reinforce visual instructions.

**Figure 5:**
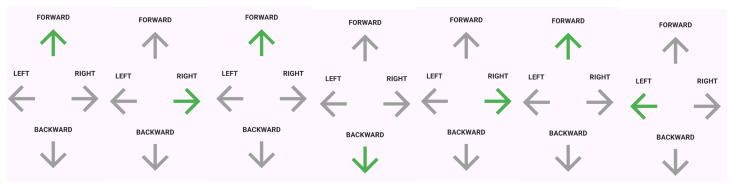
User interface.

The core of the Flutter application is encapsulated within the MainPage, a StatefulWidget that orchestrates the lifecycle of the evacuation guidance view. The layout employs a responsive hierarchy rooted in a Scaffold widget, which provides the fundamental material design visual structure. The main body is centered and arranged vertically using a Column widget, as depicted in Figure 5. The directional grid is structured with a forward indicator at the top, a middle row containing left and right indicators, and a backward indicator at the bottom. Additionally, a control interface includes a “Start” button to initiate the guidance sequence, primarily for simulation and testing.

The visual state of the directional arrows is managed dynamically by an active arrow state variable. In the active state, the arrow corresponding to the current navigational instruction is rendered in green to signify the correct path. Conversely, all inactive arrows are rendered in grey. This contrast is intentionally designed to reduce cognitive load, allowing the user to focus solely on the active direction without distraction. To further enhance situational awareness, the UI integrates with the flutter_tts engine. The system is programmed to trigger a synthesized voice command (e.g., “Forward”, “Right”) synchronously with visual state changes. This multimodal feedback mechanism ensures that users receive directional guidance even if their visual attention is momentarily diverted. The UI architecture is designed to be a passive consumer of data from backend modules. The evacuation system relies on two primary modules: the Navigation Module and the Routing Module. The Navigation Module is responsible for real-time computation of the user’s precise indoor location. Subsequently, the Routing Module uses this location data to dynamically calculate the shortest path to the nearest safe exit and determine the immediate directional vector required for the user.

### 7.2. Pathfinding Logic

The pathfinding logic, already introduced in Section 4, is encapsulated in the **DijkstraAlgorithm** class. The implementation uses Dart’s standard library collections to improve efficiency. The implementation uses the following key data structures: **GraphNode** represents a physical location with a unique ID, name, exit status, and blocked status; **Edge** represents a connection between two nodes, with a base distance and a dynamically computed current cost. **SensorData** contains real-time measurements: smoke level σ∈[0,1], crowding level κ∈[0,1], fire presence flag ϕ, and timestamp. **Graph** maintains the complete building topology with methods for path finding, cost updates, and node management.

### 7.3. Indoor Positioning and Graph Mapping Implementation

The positioning logic is encapsulated within a dedicated background isolate. The main thread communicates with the positioning isolate via asynchronous message passing using SendPort and ReceivePort with the following information: an input list of detected AccessPoint objects, containing known coordinates (x,y) and estimated distances derived from the Received Signal Strength Indicator (RSSI), an output PositioningResult object containing the computed device coordinates, and the identifier of the nearest graph edge.

The core positioning logic employs a 2D trilateration algorithm, already presented in Section 6. Given distances $r_{1},r_{2},r_{3}$ from three known access points $P_{1},P_{2},P_{3}$, the device’s position (x,y) is determined by solving the intersection of three circles defined by

(13) $${\left(x-x_{i}\right)}^{2}+{\left(y-y_{i}\right)}^{2}=r_{i}^{2}\;\mathrm{for}\;i=1,2,3$$

The implementation linearizes these equations by subtracting them pairwise to eliminate the quadratic terms, resulting in a system of linear equations of the form Ax+By=C, which is solved using Cramer’s rule or substitution. Once the coordinate (x,y) is determined, the system identifies the user’s location within the building’s navigational context. The building is modeled as a graph G(V,E), where the nodes *V* represent junctions or access points and the edges *E* represent walkable corridors. The algorithm iterates through all edges in the graph. For each edge defined by endpoints *A* and *B*, it calculates the perpendicular distance from the user’s position *P* to the line segment $\bar{AB}$. The edge minimizing this distance is selected as the user’s current location. This projection logic handles cases in which the projection falls outside the segment endpoints by clamping it to the nearest endpoint.

## 8. Simulation-Based Evaluation of the Smart Indoor Sensor Network

To comprehensively evaluate the system’s performance and obtain a complete view of its behavior, simulations of the communication system were conducted. The simulation results were also incorporated into the performance assessment, as they help identify and prevent potential issues before deployment.

For the building previously described (Figure 3), the design, implementation, and simulation-based evaluation of an indoor sensor network to model a multi-room building environment are presented. The proposed system is implemented and validated in the ns-3 network simulator and integrates heterogeneous wireless sensor nodes, Wi-Fi access infrastructure, and a mobile user running a monitoring application. The primary objective is to analyze communication performance, user-centric Quality of Service (QoS) metrics, and network behavior under spatial mobility. First, the simulation consists of two rooms, a hallway, one server floor, and a building server. Second, an extended version of the simulation scenario was scaled to represent a whole smart office building. All simulations were executed in a VMware virtualized environment, running Ubuntu Linux with ns-3 version 3.30, thereby ensuring reproductibility and isolation from host-level variability. A second case study was conducted to introduce mobile users into the simulation, allowing them to receive information as they walk through the simulation area.

### 8.1. Simulation Environment and Scaled Building Architecture

**Figure 6:**
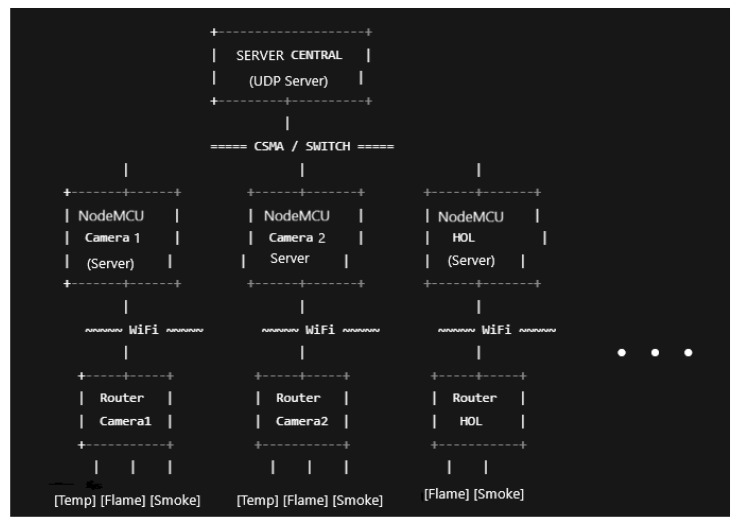
The network architecture.

We use a virtual machine to provide a controlled, deterministic execution platform, particularly suitable for large-scale discrete-event simulations. Additionally, we selected the Ubuntu operating system for its native support for ns-3 and its associated build system (waf), and the ns-3.30 simulation environment for its stability and mature support for Wi-Fi, CSMA, mobility, and application-level instrumentation, all of which were essential for modeling the proposed heterogeneous indoor network. The extended model represents a single-floor building comprising 25 rooms (laboratories and offices), with hallways (10 Wi-Fi networks), four floor servers, and a central server, thereby significantly increasing network scale and complexity. The connection between sensors and the AP is simulated via Wi-Fi, and the connections among the APs, switches, and the central server are simulated via wired Ethernet. Figure 6 represents the architecture of the network components.

1. **Rooms**. Each room is equipped with
  - One Wi-Fi router (access point).
  - One NodeMCU-like edge server.
  - Three wired sensor nodes (temperature, CO_2_, and smoke).
  In total, the room subsystem includes 25 NodeMCU nodes and 75 sensor nodes. Sensor data are periodically collected by the local NodeMCU server, enabling edge-level processing and reducing unnecessary upstream traffic.
2. **Hallways**. The hall area is modeled as a high-density sensing zone and contains
  - One Wi-Fi router (access point).
  - One NodeMCU-like edge server.
  - Two wired sensor nodes (temperature and smoke).
  In total, the hallway subsystem includes 10 NodeMCU nodes and 20 sensor nodes.
3. **Floor server**. This server consists of a switch. In total, this subsystem consists of four units.
4. **Building server**. The central server, which receives all the sensor data from the network, can monitor each component.

With this configuration, we include real-world deployments where shared spaces require greater sensing granularity due to higher occupancy and safety requirements. To ensure scalability and maintain realistic traffic aggregation, the network architecture was adopted with a hierarchical, multi-tier design:

- **Low-level Tier**. NodeMCU nodes function as local servers that aggregate sensor data from rooms and hallways.
- **Floor Server Tier**. Four dedicated floor server nodes are deployed, each responsible for a specific spatial zone of the floor. Rooms and hall NodeMCU nodes are assigned to the nearest floor server. Connectivity between NodeMCU nodes and floor servers is modeled using wired CSMA links that represent Ethernet-based building infrastructure.
- **Building Server Tier**. A three-floor building server collects and processes data forwarded from the floor servers. This node represents centralized monitoring, long-term storage, and analytics services.

### 8.2. Modelling the Traffic and the Data Flow

**Figure 7:**
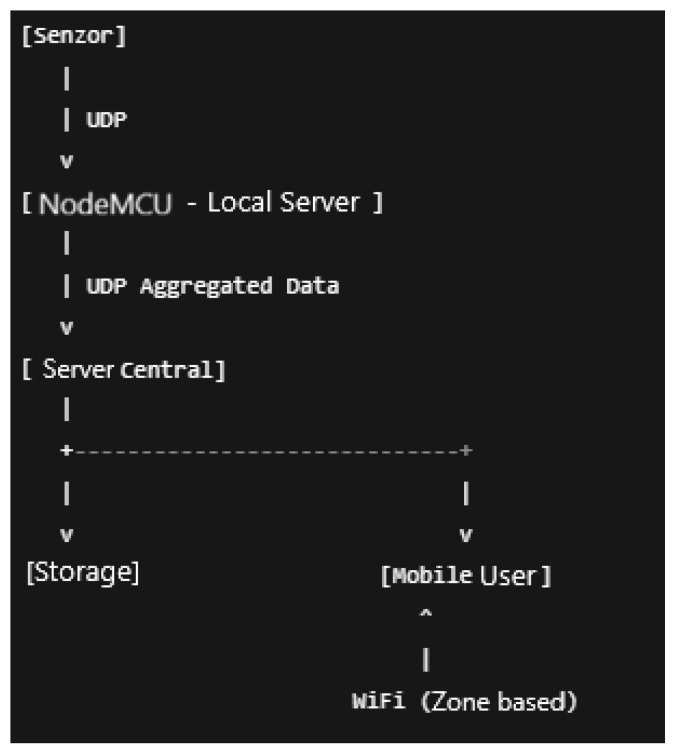
The sensors’ data flow.

Sensor nodes are programmed to generate periodic UDP traffic toward their respective NodeMCU servers. Each NodeMCU forwards aggregated data upstream to its assigned floor server, which then relays the summary to the building server. The data flow considered for the sensors is presented in Figure 7.

**Figure 8:**
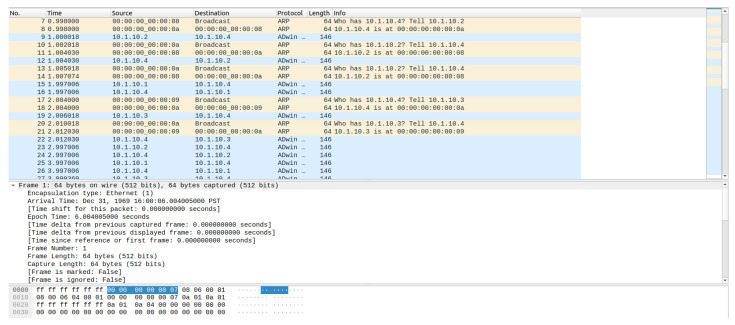
The captured traffic of the server.

During the simulation, we intended to study the traffic aggregation efficiency, packet loss across tiers, and latency accumulation from edge to cloud-like services. The increased node count enables stress testing of the wireless access points, CSMA backbone, and application-level buffering mechanisms. The extended simulation retains all instrumentation mechanisms introduced in the baseline model. Custom logging is used to track sensor transmissions, server receptions, and inter-tier forwarding events. Packet capture (PCAP) files are generated at multiple aggregation points, and CSV files are created from simulation data on Packet Delivery Ratios, end-to-end delays, jitter evolution, and energy consumption estimates at edge and user nodes. The model’s scalability enables comparative evaluation and highlights performance bottlenecks and design trade-offs. Captured network traffic for each active node can be visualized in Wireshark. A capture of the server traffic is presented in Figure 8. Table 1 represents an example of the data traffic for the sensors and how the data is captured.

**Figure 9:**
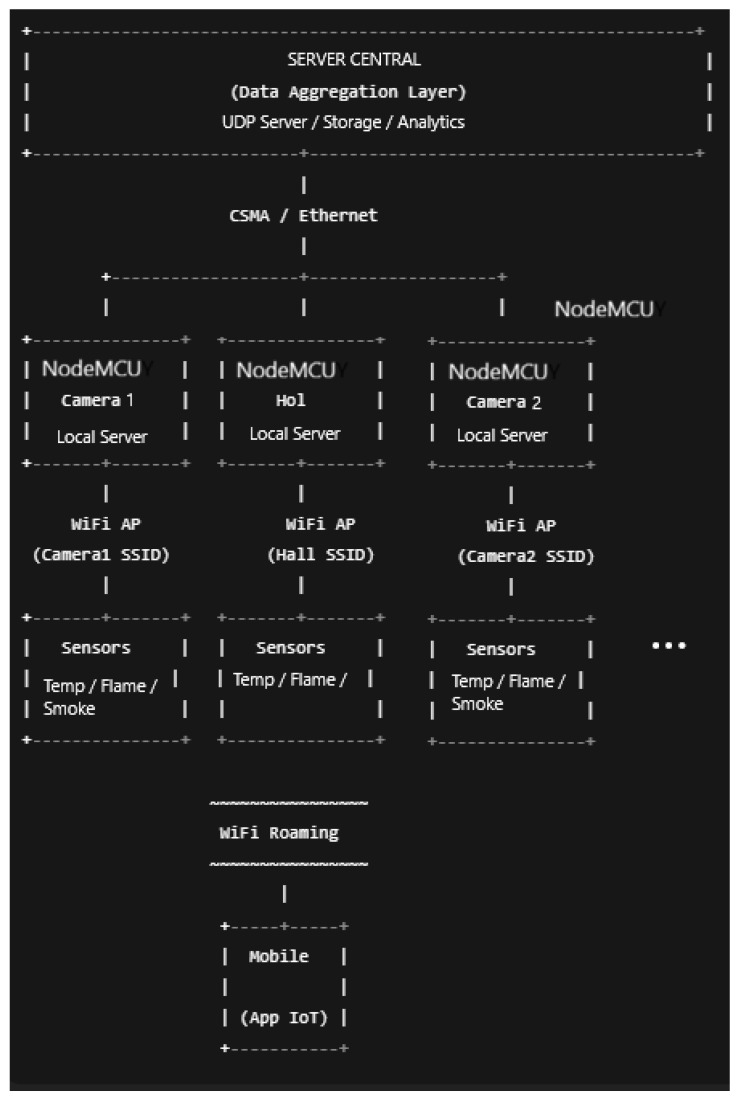
The extended network architecture.

During the simulation, we introduced a new type of mobile node, the user, which can move within the simulated map and receive messages. These messages are delivered through the nearby AP. Figure 9 presents the network architecture, including a mobile user.

During simulation, we monitor and compute several parameters to assess the Quality of Service of the selected network topology. Table 2 presents the Packet Delivery Ratio (PDR) for the system’s main communication segments. The results indicate a consistently high delivery reliability throughout the network. Communications between sensors and their associated NodeMCU nodes in both rooms and the hallway achieve PDR values above 98%, demonstrating the effectiveness of short-range, single-hop communication in dense indoor environments. A slight decrease in PDR is observed on the NodeMCU-to-floor server links (97.24%), which can be attributed to increased traffic aggregation and queue contention at intermediate nodes. Nevertheless, this value remains within acceptable limits for real-time monitoring applications. The highest reliability is observed on the floor server-to-building server segment, reflecting the stable, low-interference backbone communication between higher-tier nodes. The lowest PDR (94.22%) occurs for sensor-to-mobile user traffic. This behavior is expected due to user mobility, dynamic link conditions, and handover effects, which introduce additional packet losses compared to static infrastructure-based communication.

1. **Packet Delivery Ratio**. The mobile user experiences the lowest PDR; handover-like effects and transient disconnections are the cause.
2. **End-to-End Delay**. The considered measurement path is *Sensor* →*NodeMCU*→*Floor Server*→ *Building Server/Mobile User*.
  - Delay components.
  - Wi-Fi access delay.
  - CSMA queuing delay.
  - Application layer processing.
  - Mobility-induced reassociation delay (user).
  Mobility introduces delay spikes, especially during transitions between the hall and rooms. The end-to-end delay statistics summarized in Table 3 highlight the latency performance of the hierarchical network architecture. Direct sensor-to-NodeMCU communication exhibits the lowest average delay (3.2 ms), confirming that edge processing significantly reduces transmission latency. As data traverses additional network layers, the average delay increases gradually. Sensor-to-floor server and sensor-to-building server paths introduce moderate latency increases due to multi-hop forwarding and processing at intermediate nodes. However, even the longest static path maintains a mean delay below 10 ms, which is suitable for delay-sensitive monitoring and alerting applications. The highest delays are observed in communications involving a mobile user, particularly under mobility conditions. The increased maximum delay (42.6 ms) reflects transient disconnections and re-routing events, yet the average delay remains acceptable for non-critical user-facing services such as visualization and situational awareness.
3. **Jitter Evolution**. Jitter remains low and bounded during steady-state operation, but increases temporarily during mobility events. Table 4 illustrates the temporal evolution of packet jitter experienced by the mobile user. The results show that jitter values remain low across all time intervals, generally below 2 ms. Slight increases in jitter are correlated with user movement between rooms and hallways, where signal quality and routing paths change. The limited jitter variation demonstrates the network’s stability despite mobility and traffic aggregation, indicating that the scheduling and buffering mechanisms effectively mitigate delay fluctuations. Such characteristics are essential for applications involving real-time data streaming or periodic sensor updates.
4. **Energy Consumption Estimates**. We calculate the per-packet energy for the energy models. Energy consumption scales primarily with packet aggregation role, validating the efficiency of the hierarchical architecture. The energy consumption results are divided between sensing devices and higher-tier network nodes. Table 5 shows that individual sensors deployed in rooms consume slightly more energy than those in the hall, primarily due to higher reporting frequencies and increased data generation. Table 6 highlights the energy cost of computation and communication at the edge and server levels. NodeMCU nodes exhibit moderate energy consumption, validating their suitability as edge processing units in large-scale indoor deployments. Floor and building servers naturally entail higher energy costs due to traffic aggregation and centralized processing. The mobile user device consumes the most energy due to continuous reception, processing, and mobility-related signaling. This result emphasizes the importance of offloading computation to edge and server nodes to prolong battery life at the user end.

During simulation, the obtained results show that the proposed architecture achieves Packet Delivery Ratios above 97%, with average end-to-end delays below 10 ms for infrastructure traffic and bounded jitter during user mobility.

## 9. Experimental Validation and Results

**Figure 10:**
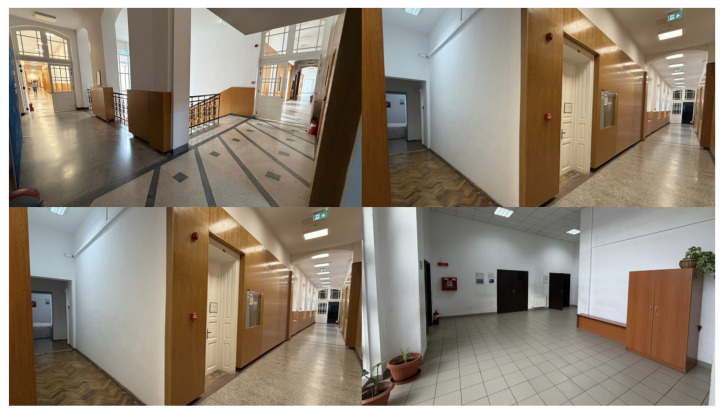
Real-world reference images for the simulated node locations.

The following three scenarios simulate critical emergencies where a fire breaks out near the optimal path, accompanied by severe crowding and smoke in adjacent areas. The tests aim to evaluate the system’s ability to handle absolute blockages (e.g., fire) and high-cost penalties (e.g., crowd/smoke) simultaneously. Figure 10 includes representative photographs of the existing building, corresponding to the nodes presented below in the scenarios.

### 9.1. Scenario 1

#### 9.1.1. Phase 1: Initial Path Calculation (t<10 s)

With uniform edge costs (1.0), the system identified the shortest path from the Start Position (Node 17) to the nearest exit:

- **Route:** Node 17 → Node 12 → Node 8 → Exit 1.
- **Total Cost:** 3.00.
- **Selected Exit:** Exit 1.

#### 9.1.2. Phase 2: Emergency Response (t=10 s)

At t=10 s, the emergency sensors triggered the following updates:

- **Node 10:** Penalty applied for Fire (Blocked).
- **Node 8:** Penalties applied for Smoke (0.8) and Crowding (0.9).

- **Initial State:** All paths are clear with a uniform base cost of 1.0 per edge.
- **Event Trigger (**t=10 **s):**
  − **Node 10:** Signals Fire (Complete Blockage).
  − **Node 8:** Signals High Smoke (0.8) and Very High Crowding (0.9).
- **Objective:** The system must identify Node 10 as impassable and Node 8 as highly dangerous, and then find an alternative route that avoids these risks.

**Figure 11:**
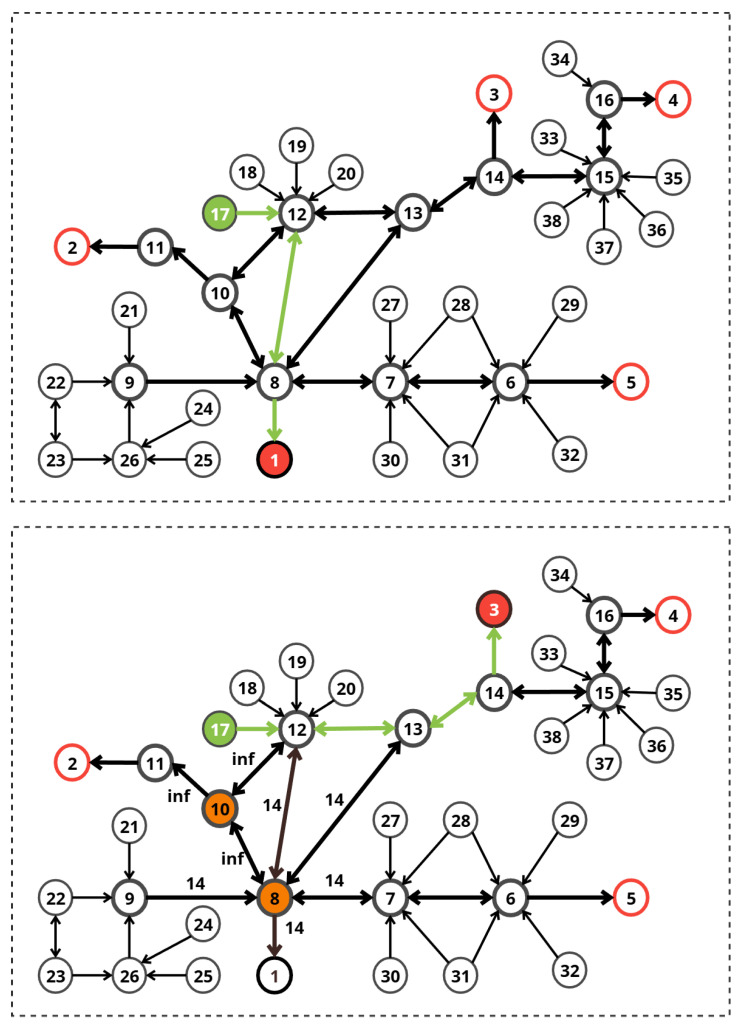
Evacuation scenario 1.

Figure 11 presents the two phases of Scenario 1. Red circles represent exits, the green circle represents the source node, the initial and modified paths are shown with green arrows, while the orange circles represent penalized nodes.

Table 7 lists the edges connected to the affected nodes in Scenario 1. Edges connected to Node 10 (Fire) incur the extreme fire penalty, effectively blocking them.

The dynamic Dijkstra algorithm recomputed the route. Since Node 8 (part of the original path) became high-cost and Node 10 (a potential alternative) was blocked, the system selected a completely different path:

- **New Route:** Node 17 → Node 12 → Node 13 → Node 14 → Exit 3.
- **New Total Cost:** 4.
- **Selected Exit:** Exit 3.

The system successfully navigated a complex hazard configuration. Node 10 was excluded from the search space entirely. Although Node 8 was not strictly blocked, the high penalties associated with smoke and crowding made it a suboptimal choice. The system identified the next-best alternative (Exit 3) with a minimal cost increase (from 3 to 4), ensuring evacuee safety without an excessive detour.

### 9.2. Scenario 2

#### 9.2.1. Phase 1: Initial Path Calculation (t<10 s)

With uniform edge costs (1.0), the system identified the shortest path from the Start Position (Node 8) to the nearest exit:

- **Route:** Node 8 → Exit 1.
- **Total Cost:** 1.00.
- **Selected Exit:** Exit 1.

#### 9.2.2. Phase 2: Emergency Response (t=10 s)

At t=10 s, the emergency sensors triggered the following updates:

- **Node 1:** Penalty applied for Fire (Blocked).
- **Node 11 and 14:** Penalties applied for Smoke (0.8) and Crowding (0.8).

- **Initial State:** All paths are clear with a uniform base cost of 1.0 per edge.
- **Event Trigger (t=10 s):**
  − **Node 1:** Signals Fire (Complete Blockage).
  − **Nodes 11 and 14:** Signals High Smoke (0.8) and Very High Crowding (0.8).
- **Objective:** The system must identify Node 1 as blocked and Node 11 and 14 as highly dangerous, finding an alternative route that avoids these risks.

Table 8 lists the edges connected to the affected nodes in Scenario 2. Edges connected to Node 1 (Fire) incur the extreme fire penalty.

**Figure 12:**
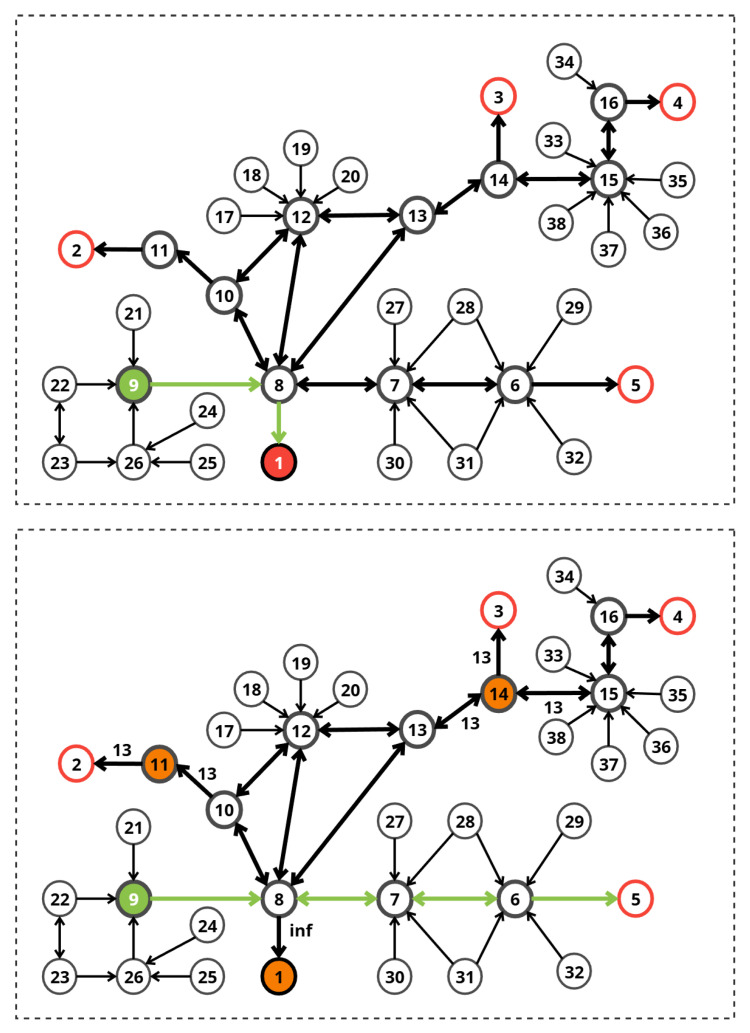
Evacuation scenario 2.

Figure 12 presents the two phases of Scenario 2. Red circles represent exits, the green circle represents the source node, the initial and modified paths are shown with green arrows, while the orange circles represent penalized nodes.

The dynamic Dijkstra algorithm recomputed the route. Since Nodes 11 and 14 became high-cost and Node 1 (the shortest exit) was blocked, the system selected the following shortest path:

- **New Route:** Node 9 → Node 8 → Node 7 → Node 6 → Exit 5.
- **New Total Cost:** 4.
- **Selected Exit:** Exit 5.

The system successfully navigated a complex hazard configuration. Node 10 was excluded from the search space entirely. The system identified the best alternative (Exit 5), ensuring evacuee safety without an excessive detour.

### 9.3. Scenario 3

#### 9.3.1. Phase 1: Initial Path (*t* = 0 s)

The system calculated the optimal path under normal conditions.

- **Route:** Node 8 → Exit 1.
- **Total Cost:** 1.00.

#### 9.3.2. Phase 2: Emergency Response (*t* = 10 s)

Sensors detected extreme hazards at multiple locations. The system marked these nodes as blocked or high-cost and recalculated the route.

- **Start Node:** 8.
- **Initial Conditions:** Normal, all paths clear.
- **Event (*t* = 10 s):**
  − **Exit 1 and Node 6:** Fire detected (blocked).
  − **Exit 3 and Node 11:** Crowding detected.

**Figure 13:**
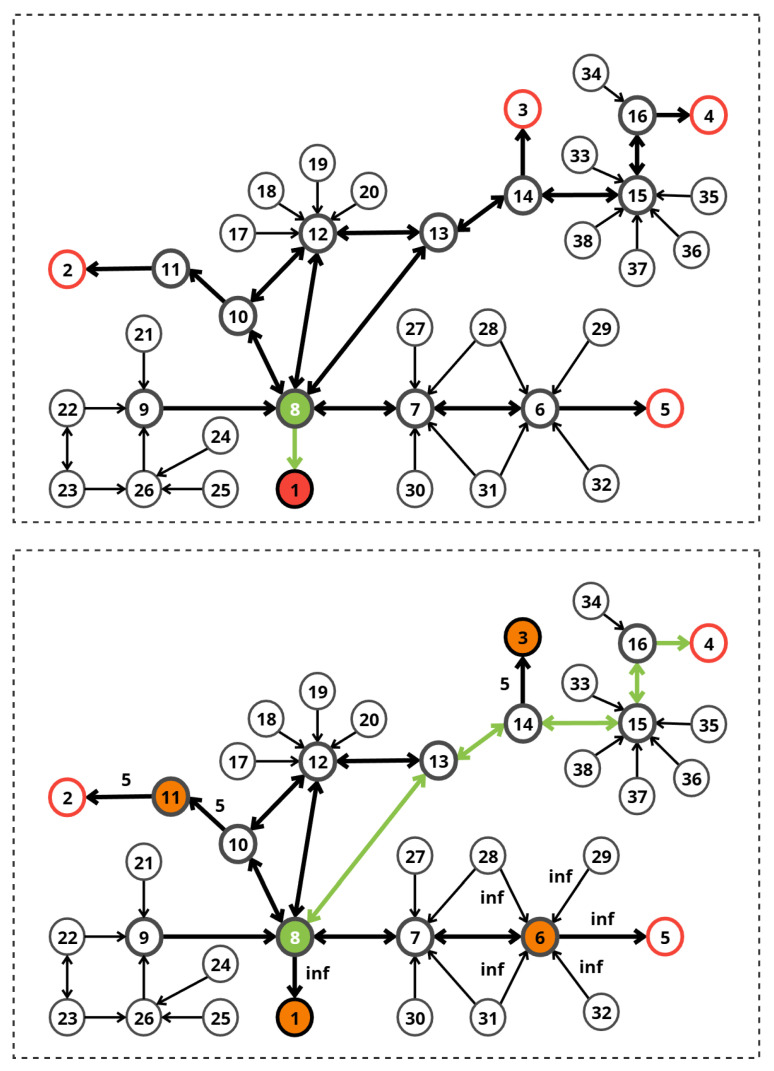
Evacuation scenario 3.

Figure 13 presents the two phases of Scenario 3. Red circles represent exits, the green circle represents the source node, the initial and modified paths are shown with green arrows, while the orange circles represent penalized nodes.

Table 9 lists the edges connected to the affected nodes in Scenario 3. Edges connected to Node 1 (Fire) incur the extreme fire penalty.

The simulation demonstrated the system’s ability to find the single remaining safe path in a highly constrained environment. The algorithm correctly evaluated and rejected paths leading to Exits 1, 2, 3, and 5 due to blockages at the exits or at critical intermediate nodes (Nodes 11 and 6). Exit 4 was identified as the only reachable safe zone. The selected path (8–13–14–15–16–4) successfully bypasses all hazard zones. Note that while Node 14 connects to the blocked Node 3, Node 14 itself was not blocked, allowing safe transit to Node 15.

The pathfinding algorithm’s performance on the mobile emulator was computed as the average of 10 runs per scenario. The initial path calculation took an average of 100 ms, and the recalculation took an average of 150 ms. The algorithm is highly efficient and suitable for real-time use on mobile devices.

To evaluate the system’s performance, we conducted a set of experimental scenarios in the complex multi-exit building described in the article, with a visually impaired participant. Table 10 summarizes four experimental scenarios, with results reported for each case. In Trial 2, the initially selected route became unsafe due to the risk of fire spread, requiring rerouting during the evacuation simulation.

## 10. Conclusions

This research presented a comprehensive mobile evacuation guidance system designed to address the critical safety gaps faced by visually impaired individuals during fire emergencies. By shifting the computational burden from vulnerable building infrastructure to the user’s mobile device, the proposed decentralized architecture ensures operational continuity even when central networks fail. The system integrates real-time environmental monitoring with personalized navigation, providing a robust safety net that adapts to the unpredictable evolution of fires.

The technical core of the solution relies on a modified Dijkstra’s algorithm that goes beyond static pathfinding. By incorporating dynamic edge weights derived from real-time sensor data—including smoke density, crowd congestion, and fire proximity—the system effectively models the changing risk landscape. This is complemented by a Wi-Fi RSSI-based positioning system that, despite the inherent challenges of indoor signal propagation, provides sufficient accuracy for corridor-level navigation. The integration of these components into a cross-platform Flutter application demonstrates the practical feasibility of performing complex, life-critical computations directly on consumer hardware with negligible latency.

Experimental validation across multiple simulated scenarios confirmed the system’s ability to make life-saving decisions in real time. In tests involving sudden blockages and rapidly spreading hazards, the routing engine successfully identified safe alternatives, diverting users from compromised exits to viable safe zones with minimal delay. Performance evaluation on mobile emulators showed that initial path calculations average 100 ms, while critical recalculations occur in just 150 ms, comfortably meeting the sub-second response times required for emergency guidance.

The simulation conducted using the ns-3 platform confirms that the proposed multi-tier architecture achieves high reliability, low latency, stable temporal behavior, and efficient energy use. Separating sensing, edge processing, and centralized management enables scalable deployment while maintaining quality-of-service guarantees.

Privacy and security were foundational to the system’s design, not afterthoughts. The architecture strictly adheres to privacy-by-design principles, ensuring that sensitive user location data never leaves the device. By relying on broadcast-only sensor networks and local processing, the system eliminates the risks associated with centralized tracking while maintaining full functionality. This approach not only protects user privacy but also enhances system scalability, as the network does not become a bottleneck during mass evacuations.

Future work will focus on refining the user interaction model, specifically by implementing a more granular directional guidance system using the Flutter framework to ensure cross-platform compatibility. The proposed implementation prioritizes inclusive design, aiming to expand the system’s utility beyond visual impairments. Future iterations will incorporate redundant sensory cues, such as enhanced haptic patterns and visual strobing, to support deaf and hard-of-hearing individuals. Additionally, further research is planned to validate the system in larger, multi-story complexes and to explore integrating sensor fusion techniques [47,48], combining Wi-Fi RSSI with inertial odometry to improve positioning stability in complex environments [49,50].

## 11. Limitations and Practical Considerations

While the proposed system demonstrates significant potential to enhance the safety of individuals with impairments during fire evacuations, several limitations and practical challenges must be addressed to ensure robust deployment in real-world scenarios. It is important to highlight, however, that the current decentralized architecture and cross-platform implementation provide a scientifically sound methodological foundation. By shifting the computational load to the user’s device, the system reduces latency and the dependence on central processing servers, a critical step toward resilience. The successful integration of real-time sensor data into a modified Dijkstra’s algorithm validates the core concept of dynamic routing. Nevertheless, to bridge the gap between this promising proof of concept and a fully deployable industrial solution, this section critically examines the system’s reliance on infrastructure and the current scope of its evacuation logic.

### 11.1. Infrastructure Dependency and Resilience

A critical limitation of the current implementation is its reliance on the building’s Wi-Fi infrastructure for both indoor positioning (RSSI-based) and data transmission. In a severe fire incident, power outages or structural damage may compromise the Wi-Fi network, potentially rendering the localization and real-time update mechanisms inoperable. This contradicts the goal of a fully resilient, decentralized system.

To mitigate this risk, future iterations of the system must incorporate multimodal fallback strategies. Specifically, integrating Bluetooth Low Energy (BLE) beacons, which can operate on battery power independent of the building’s main electrical supply, would provide a robust alternative for localization when Wi-Fi is unavailable. Additionally, leveraging inertial measurement units (IMUs) on the user’s device (accelerometers and gyroscopes) could enable dead-reckoning navigation in a degraded mode, allowing the application to maintain a position estimate even without external signals. Furthermore, exploring opportunistic device-to-device (D2D) communication protocols could allow users’ devices to share hazard information directly, creating a mesh network that functions without a central server or access points.

### 11.2. Evacuation Logic and Decision Support

The current routing algorithm focuses primarily on finding the optimal path to an exit. However, this approach simplifies the complex decision-making required in a fire emergency, particularly for mobility-impaired users. The system currently lacks a comprehensive Risk Assessment step to determine whether immediate evacuation is indeed the safest option compared to a “defend-in-place” strategy.

For users with severe mobility limitations, attempting to traverse a smoke-filled corridor might pose a greater risk than remaining in a designated fire-safe compartment. To address this, future developments will expand the decision support logic to include a predictive risk protocol. This would involve integrating a simplified fire dynamics model to forecast the spread of smoke and heat over a short time horizon (e.g., the next 5–10 min). By making path costs temporally aware, the system could recommend a “shelter-in-place” strategy if all viable evacuation routes are predicted to become hazardous before the user can traverse them. The decision logic should explicitly evaluate the safety of the current zone against the predicted conditions of the evacuation path, and provide recommendations such as “Evacuate via Route A, Move to Refuge Area B,” or “Remain in Safe Zone.”

### 11.3. Network Degradation

While the experimental validation demonstrated efficiency gains, the tests assumed a functioning network environment. Real-world fire scenarios often involve significant signal interference and network congestion. Future validation efforts will include simulations of network degradation to quantify the system’s performance limits under packet loss and high-latency conditions, ensuring that the fail-safe mechanisms are triggered reliably when communication quality drops below a critical threshold.

## Figures and Tables

**Table 1:** Sensors’ data traffic.

Time	Zone	Seonsor ID	Destination IP
1	Room 1	0	10.1.1.1
2	Room 2	0	10.1.2.1
2	Hall 1	0	10.1.3.1
3	Room 1	1	10.1.1.1
3	Room 2	1	10.1.2.1
4	Hall 2	0	10.1.4.1
4	Room 1	2	10.1.1.1
	…		

**Table 2:** Packet Delivery Ratio (PDR) for different network segments.

Network Segment	Packets Sent	Packets Received	PDR (%)
Sensors → NodeMCU (Rooms)	18,750	18,520	98.77
Sensors → NodeMCU (Hall)	4200	4135	98.45
NodeMCU → Floor Server	7600	7390	97.24
Floor Server → Building Server	2400	2365	98.54
Sensors → Mobile User	3200	3015	94.22

**Table 3:** End-to-end delay statistics.

Traffic Path	Mean (ms)	Min (ms)	Max (ms)
Sensor → NodeMCU	3.2	1.9	6.1
Sensor → Floor Server	6.8	4.5	12.3
Sensor → Building Server	9.5	6.0	18.7
Sensor → Mobile User (Static)	8.9	6.1	15.4
Sensor → Mobile User (Mobile)	14.7	7.2	42.6

**Table 4:** Jitter evolution for the mobile user.

Time Interval (s)	User Location	Average Jitter (ms)
0–5	Hall	1.1
5–10	Camera 1	1.6
10–15	Hall	1.3
15–20	Camera 2	1.8
20–25	Hall	1.2

**Table 5:** Average energy consumption per sensor.

Zone	Number of Sensors	Energy per Sensor (mJ)
Rooms	75	9.8
Hall	20	7.2

**Table 6:** Energy consumption of edge and user nodes.

Node Type	Count	Avg Energy per Node (mJ)
Room NodeMCU	25	42.5
Hall NodeMCU	10	38.1
Floor Server	4	61.4
Building Server	1	74.9
Mobile User	1	150.8

**Table 7:** Edge costs for Node 8 and Node 10 at t=10 s.

From	To	Initial Cost	Cost/Status
1	8	1	14
7	8	1	14
8	9	1	14
8	12	1	14
8	13	1	14
8	10	1	inf
10	11	1	inf
10	12	1	inf

**Table 8:** Edge costs for Nodes 1, 8, and 10 at t=10 s.

From	To	Initial Cost	Cost/Status
1	8	1	inf
11	2	1	13
10	11	1	13
3	14	1	13
14	15	1	13
13	14	1	13

**Table 9:** Edge costs for Nodes 1, 11, 6, and 3 at t=10 s.

From	To	Initial Cost	Cost/Status
1	8	1	inf
10	11	1	5
2	11	1	5
3	14	1	5
5	6	1	inf
6	7	1	inf
6	28	1	inf
6	6	31	inf

**Table 10:** Evacuation time for different trials.

Trial	Evacuation Time Without Guidance (s)	Evacuation Time With Guidance (s)
1	145.5	121.3
2	280.3	114.8
3	113	88.7
4	315.6	278

## Data Availability

The original contributions presented in this study are included in the article. Further inquiries can be directed to the corresponding authors.

## Abbreviations

    The following abbreviations are used in this manuscript:

BLE	Bluetooth Low Energy
IMUs	Inertial Measurement Units
RSSI	Received Signal Strength Indicator
WLS	Weighted Least Squares
GPS	Global Positioning System
D2D	device-to-device
QoS	Quality of Service
UWB	Ultra-Wideband
SDK	Software Development Kit
AR	Augumented Reality
RFID	Radio Frequency Identification
DSS	Decision Support System
CRC	Cyclic Redundacy Check
CBC-MAC	Cipher Block Chaining Message Authentication Code

## Appendix A

**Algorithm A1** RSSI-Based Position Estimation				
1:	**procedure** EstimatePosition(access_points,rssi_readings)			
2:		distances←[]		▹ Estimated distances to each AP
3:		weights←[]		▹ Confidence weights for each measurement
4:		**for** i←1 to *n* **do**		▹ Process each access point
5:			$d_{i}\leftarrow 10^{\left(RSSI_{0}-rssi_{i}\right)/\left(10\cdot \eta \right)}$	▹ Convert RSSI to distance
6:			$w_{i}\leftarrow rssi_{i}^{2}$	▹ Weight by signal strength
7:			distances.append($d_{i}$)	
8:			weights.append($w_{i}$)	
9:		**end for**		
10:		(x,y)← WeightedLeastSquares(access_points,distances,weights)		
11:		**return** (x,y)		
12:	**end procedure**			

**Algorithm A2** Best Exit Selection Algorithm
1: **procedure** FindBestExit($G,s,V_{exit},c$) 2: $P_{best}\leftarrow \mathrm{null}$ 3: $c_{min}\leftarrow \infty$ 4: **for** each $e\in V_{exit}$ **do** 5: P← FindShortestPath(G,s,e,c) 6: $c_{P}\leftarrow$ TotalCost(P,c) 7: **if** $c_{P}<c_{min}$ **then** 8: $c_{min}\leftarrow c_{P}$ 9: $P_{best}\leftarrow P$ 10: **end if** 11: **end for** 12: **return** $P_{best}$ 13: **end procedure**

**Algorithm A3** Dynamic Cost Update Algorithm					
1:	**procedure** UpdateDynamicCosts(G,S)				
2:		**for** each $e_{ij}\in E$ **do**			
3:			$c_{ij}\leftarrow d_{ij}$		▹ Reset to base distance
4:		**end for**			
5:		**for** each $e_{ij}\in E$ **do**			
6:			**if** $S_{i}$ exists or $S_{j}$ exists **then**		
7:				${\bar{\sigma }}_{ij}\leftarrow$ Average smoke level from $S_{i},S_{j}$	
8:				${\bar{\kappa }}_{ij}\leftarrow$ Average crowding from $S_{i},S_{j}$	
9:				$c_{ij}\leftarrow c_{ij}\cdot \left(1+5{\bar{\sigma }}_{ij}\right)+10{\bar{\kappa }}_{ij}$	
10:			**end if**		
11:			**if** $S_{i}.\phi =\mathrm{true}$ or $S_{j}.\phi =\mathrm{true}$ **then**		
12:				$c_{ij}\leftarrow inf$	
13:			**end if**		
14:		**end for**			
15:	**end procedure**				

**Algorithm A4** Real-Time Evacuation System					
1:	**procedure** EvacuationSystem(G,s,Δt)				
2:		$P_{current}\leftarrow$ FindBestExit($G,s,V_{exit},c$)			
3:		Display path $P_{current}$			
4:		**while** not at exit **do**			
5:			Wait(Δt)		▹Δt=5 s
6:			S← CollectSensorData()		
7:			UpdateDynamicCosts(G,S)		
8:			$P_{new}\leftarrow$ FindBestExit($G,s,V_{exit},c$)		
9:			**if** $P_{new}\neq P_{current}$ **then**		
10:				Display “Route updated!”	
11:				$P_{current}\leftarrow P_{new}$	
12:				DirectionalGuidanceFor $P_{current}$	
13:			**end if**		
14:		**end for**			
15:	**end procedure**				
